# Mapping the relationship between atopic dermatitis and gut microbiota: a bibliometric analysis, 2014–2023

**DOI:** 10.3389/fmicb.2024.1400657

**Published:** 2024-09-04

**Authors:** Yilin Wang, Bingkun Wang, Shiyou Sun, Zhongzhi Wang

**Affiliations:** ^1^Department of Dermatology, The 83rd Group Army Hospital of the PLA, Xinxiang, China; ^2^Department of Dermatology, Shanghai Fourth People 's Hospital, Tongji University School of Medicine, Shanghai, China

**Keywords:** atopic dermatitis, gut microbiota, bibliometric analysis, trends, hotspots

## Abstract

**Background:**

Atopic dermatitis (AD) is a chronic inflammatory skin condition affecting a significant portion of the population, with prevalence rates of 25% in children and 7–10% in adults. AD not only poses physical challenges but also profoundly impacts patients’ mental well-being and quality of life. The stability of gut microbiota is crucial for overall health and can influence AD progression by modulating immune function, skin barrier integrity, and neuroendocrine signaling, which may be an effective target for the prevention and treatment of AD. Thus, exploring the interactions between AD and gut microbiota, particularly in infants, can provide insights into potential preventive and therapeutic strategies. This study aimed to explore the correlation between AD and gut microbiota while providing an overview of current research trends and emerging areas of interest in this field.

**Methods:**

A comprehensive search was conducted on the Web of Science Core Collection (WOSCC) for relevant publications from January 1, 2014, to December 31, 2023. English-language articles and reviews were included. Two investigators independently screened the publications, and visual analysis was performed using CiteSpace, VOSviewer, Scimago Graphica, and Microsoft Excel software.

**Results:**

A total of 804 articles were included, showing a significant increase in publications over the past decade. The United States, Wageningen University, and University Ulsan (represented by Hong SJ) had the highest number of published papers. Nutrients was the journal with the most publications, while the Journal of Allergy and Clinical Immunology had the highest number of citations and centrality among co-cited journals. Keyword visualization analysis identified “atopic dermatitis” and “gut microbiota” as central themes. Notably, there has been a notable shift in research focus over the years, with early studies concentrating on “Fecal microbiota,” “caesarean section,” and “first 6 months,” while recent studies have highlighted the roles of “cells,” “dysbiosis,” and “prebiotics.” This shift indicates growing interest in the underlying mechanisms and potential therapeutic interventions related to the intestinal microecology in AD treatment.

**Conclusion:**

The field of AD and gut microbiota research has evolved significantly, with an increasing focus on understanding the intricate interactions between gut microbiota and AD pathogenesis. Recent years have witnessed increased interest in understanding the relationship between AD and gut microbiota, with researchers conducting extensive studies exploring various aspects of this connection. This review analyzes research trends over the past decade, highlighting trends and hotspots in the study of AD, particularly in infants, and the role of microbiota. This review serves as a valuable reference for future investigations, aiming to provide deeper insights into this burgeoning field and suggests directions for future research.

## Introduction

Atopic dermatitis (AD) is a chronic relapsing inflammatory skin disease characterized by dry skin, intense itching, and eczematous rash. It has a high incidence and affects approximately 25% of children and 7–10% of adults (Odhiambo et al., 2009; Thomsen, 2015). While AD can develop at any age, it is most commonly seen in infancy (Weidinger and Novak, 2015; Li et al., 2021). As patients age, some may experience a disappearance of clinical symptoms, while others may continue to suffer and develop conditions such as allergic asthma or allergic rhinitis (Napolitano et al., 2022). AD patients often experience severe pruritus, leading to poor sleep quality. The recurrent nature of the disease significantly impacts the mental well-being and quality of life of patients, thereby imposing a substantial burden on patients and their families (Khan et al., 2021; Ali et al., 2020).

The pathogenesis of AD is thought to be influenced by various factors, including immune dysregulation (Yang et al., 2020), compromised skin barrier function (Luger et al., 2021), dysbiosis of the skin microbiome (Hrestak et al., 2022), and environmental changes (David et al., 2017). However, these factors have not been fully explained (Kim et al., 2019). Recent research suggests that gut microbiota disorders are closely associated with IgE-related eczema, asthma, AD, and other allergic diseases (Liu et al., 2023).

Maintaining a stable gut microbiota is crucial for human health as it can impact the progression of AD by regulating the host’s immune function, skin barrier, and neuroendocrine system. A clinical study (Thursby and Juge, 2017) observed that patients with AD had a significantly higher proportion of *Staphylococcus aureus*, *Escherichia coli*, and *Clostridium* in their gut microbiota, while the proportion of *Bifidobacterium bifidum* was significantly lower compared to healthy individuals. Another prospective study (Abrahamsson et al., 2012) found that children with AD had lower diversity of gut microbiota compared to normal children, with lower proportions of *Bacteroides* at 1 month and Proteus at 12 months. Interestingly, the therapeutic potential of probiotics has shown promising results in restoring dysregulated intestinal microecology and reducing pruritus symptoms in patients (Isolauri et al., 2012).

Bibliometrics is a quantitative approach used to analyze the literature in a specific research area (Ninkov et al., 2022). By analyzing the author, keywords, research institutions, countries, published journals, and other information of the literature in the field, we can gain a deeper understanding of the research hotspots and development directions in that field. This information can then be used for strategic planning, policy formulation, and has been widely applied across various subject fields (Ninkov et al., 2022; Ellegaard and Wallin, 2015; Liu Y. et al., 2022). Despite the abundance of literature on AD and gut microbiota in recent years, there has been a lack of relevant bibliometric analysis (Saeed et al., 2022; Liu Q. et al., 2022; Fang et al., 2021; Park et al., 2021; Lee and Kim, 2022). Therefore, this study aims to fill this gap by using bibliometric methods to analyze the general information, research hotspots, and emerging trends in the field of AD and gut microbiota.

The study particularly focuses on trends and hotspots related to AD, infants, and the role of gut microbiota, providing valuable insights for future investigations and contributing to the continuous development of this research field.

## Materials and methods

### Literature search and screening

The Web of Science Core Collection (WOSCC) stands as one of the preeminent and extensively utilized scientific databases globally, renowned for its substantial content quality, rendering it the most fitting database for conducting bibliometric analyses (Ding and Yang, 2022). Hence, in the pursuit of ensuring the utmost precision and authority in the outcomes of this study, the WOSCC database was meticulously employed.

The search strategy employed encompassed the following terms: TS (Topic Search) = (“Dermatitis, Atopic” OR “Atopic Dermatitis” OR “Atopic Neurodermatitis” OR “Atopic Eczema”) and TS = (“Gastrointestinal Microbiome” OR “Gut Microbiome” OR “Gut Microflora” OR “Gut Flora” OR “Gastrointestinal Microbiota” OR “Gastric Microbiome “OR “Intestinal Microbiome” OR “Intestinal Microflora” OR “Intestinal Flora” OR “Gastrointestinal Microflora” OR “Gastrointestinal Flora” OR “Gastrointestinal Microbial Community”), spanning from 01 January 2014 to 31 December 2023. The language criterion was confined to English, and the literature type was restricted to articles and reviews.

**Figure 1:**
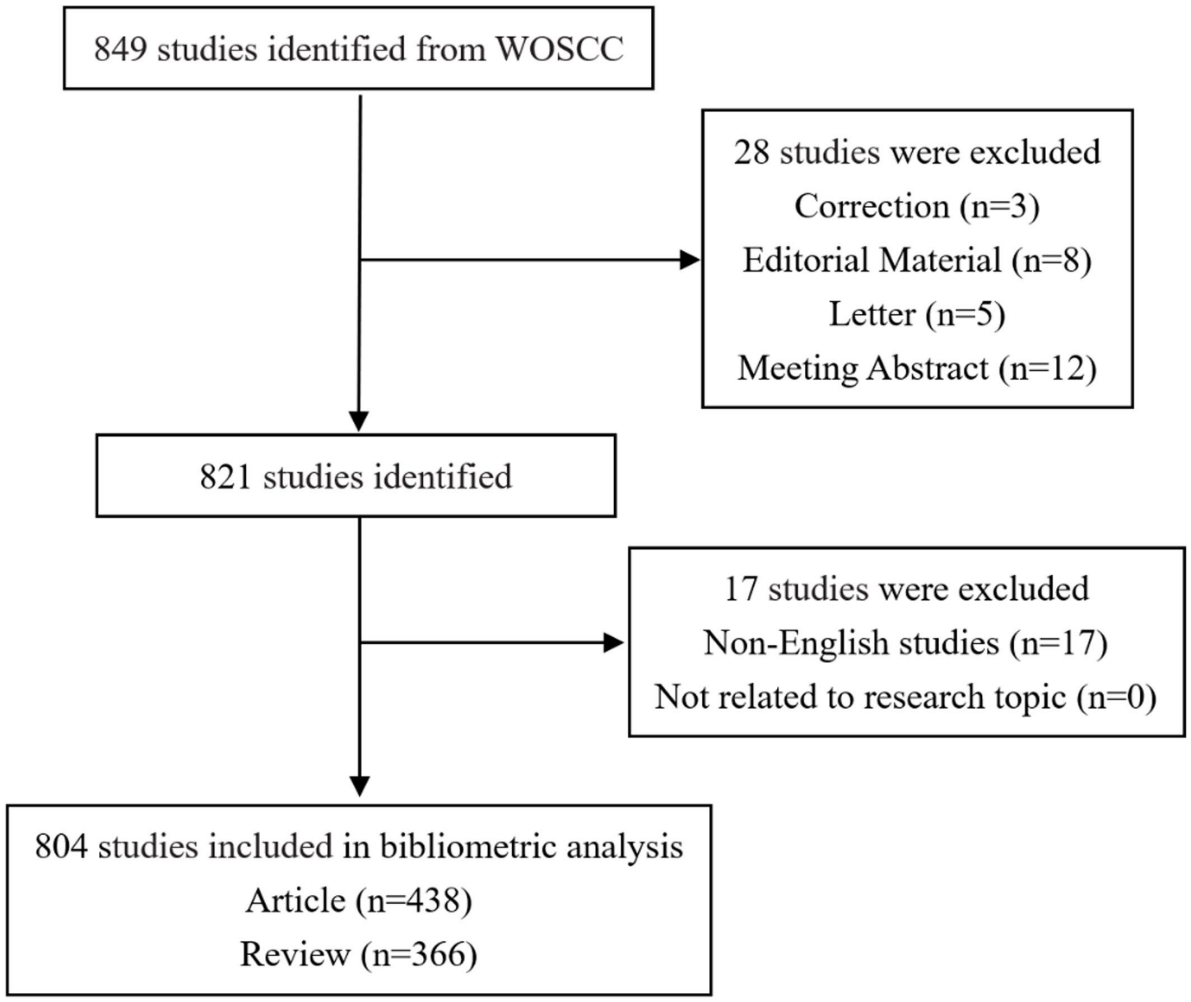
Flow chart depicting data collection strategies for AD and gut microbiota. The Web of Science Core Collection database were searched using specific terms, with the search period limited to January 1, 2014, to December 31, 2023. The inclusion criteria were papers and reviews relevant to the search terms, while letters, briefs, book reviews, and similar documents were excluded, resulting in a total of 804 articles.

Following the initial completion of the literature search, a meticulous screening process was executed by two independent investigators. In instances where discrepancies in opinions arose, a third investigator was engaged, contributing to a consultative process that ultimately led to the conclusive decision. The specific outcomes of the literature retrieval and screening process are meticulously delineated in Figure 1.

### Data analysis and visualization

After screening, WOSCC data were exported in plain text in the format of “full records and references cited,” and then imported into VOSviewer (version 1.6.20) and CiteSpace (version 6.2 R6 basic) for analysis. CiteSpace software, developed by Professor Chaomei Chen of Drexel University (Chen, 2006), and VOSviewer software, developed by Centre for Science and Technology Studies, Leiden University (Arruda et al., 2023), are both bibliometric analysis software based on the Java kernel. They are used to extract key information from WOSCC-derived data, analyze the history, hotspots, and future trends of the research field, and visualize the research results. These two software have their own advantages and disadvantages and complement each other well (Pan et al., 2018).First, we imported WOSCC data into VOSviewer software and analyzed co-authorship and countries. Then, we saved the files in “gml” format and imported them into Scimago Graphica software, which displayed more visually appealing mutual cooperation relationships between different countries. Subsequently, we used VOSviewer software to analyze co-authorship of authors and institutions, co-occurrence of keywords, co-citation of cited references, and constructed network maps. In VOSviewer’s visualization, a node represents an author, institution, keyword, or reference. The size of the node indicates the number of items, the color represents different clusters, and closer nodes indicate higher similarity. The thickness of lines between nodes reflects the degree of association between items (Van and Waltman, 2017; Van and Waltman, 2010). We also queried the H-index at WOS for the top 10 authors, institutions, journals, and countries (Hirsch, 2007).

The CiteSpace software was configured with the following settings: time span: 2014–2023, time slice: 1 year, g-index (k = 25). Cluster analysis was conducted using the log-likelihood ratio (LLR) algorithm. CiteSpace software was then utilized to generate a dual-map overlay of journals, a timeline view of keywords, and to analyze keywords with the strongest citation bursts (Chen, 2006). Finally, the annual number of publications, obtained from CiteSpace software, was inputted into Microsoft Excel 2021 to create a statistical map illustrating the trends in publication volume.

## Results

### Publication trends

**Figure 2:**
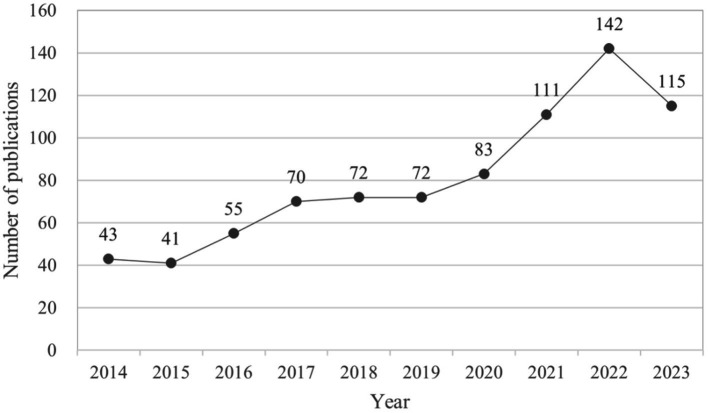
Number of publications per year focusing on AD and gut microbiota. The cumulative number of articles published on AD and microbiota from 2014 to 2023 were plotted. At the same time, an exponential function y = 36.316e^0.1307x^ (R^2^ = 0.9203, x is the year, y is the annual number of articles published) was created to model the annual publication trend, and the fit of the curve was well.

This study includes a total of 804 papers, comprising 438 articles and 366 reviews. The publication trends, illustrated in Figure 2, can be categorized into two distinct stages: prior to 2020 and subsequent to 2020. The annual publication volume demonstrates a consistent increase before 2020, followed by a notable surge from 2021 to 2023, peaking in 2022 before experiencing a decline in 2023. This trend indicates that research on AD and gut microbiota has reached a mature stage.

### Analysis of countries and institutions

The VOSviewer and Scimago Graphica software was used to visually analyze the publication regions among 72 countries, resulting in 804 AD and gut microbiota research-related articles. By setting the minimum number of publications per country to 5, we obtained a collaboration map of countries involved in AD and gut microbiota research.

In AD and gut microbiota research field, Table 1 shows the United States had the highest number of published papers (22.39%), followed by China (15.42%), South Korea (9.45%), and Italy (8.33%), among others. Percentage represents the proportion of the number of articles published by a single country to the total number of publications. The H-index, a quantitative index used to assess the number and quality of academic outputs (Hirsch, 2007), was highest in the United States, followed by China, the United Kingdom, the Netherlands, and Sweden. After the United States, the H-index of these four countries is the same, but the number of published literatures in the United Kingdom, the Netherlands, and Sweden is less than half of that in China, indicating that their research results are more recognized. This observation is consistent with the average number of citations per literature. A total of 40 countries published 5 or more papers.

**Table 1:** The top 10 countries contributing to publications of AD and gut microbiota.

Rank	Country	Number of publications	Number of citations	Citations of per article	H-index
1	USA	180	9,016	50.09	47
2	China	124	2,332	18.81	25
3	South Korea	76	1,575	20.72	20
4	Italy	67	2062	30.78	24
5	England	56	2,521	45.02	25
6	Netherlands	52	2013	38.71	25
7	Japan	51	914	17.92	16
8	Australia	46	1724	37.48	23
9	Germany	43	1,522	35.40	20
10	Sweden	34	2,155	63.38	25
11	France	34	1,275	37.50	16

**Figure 3:**
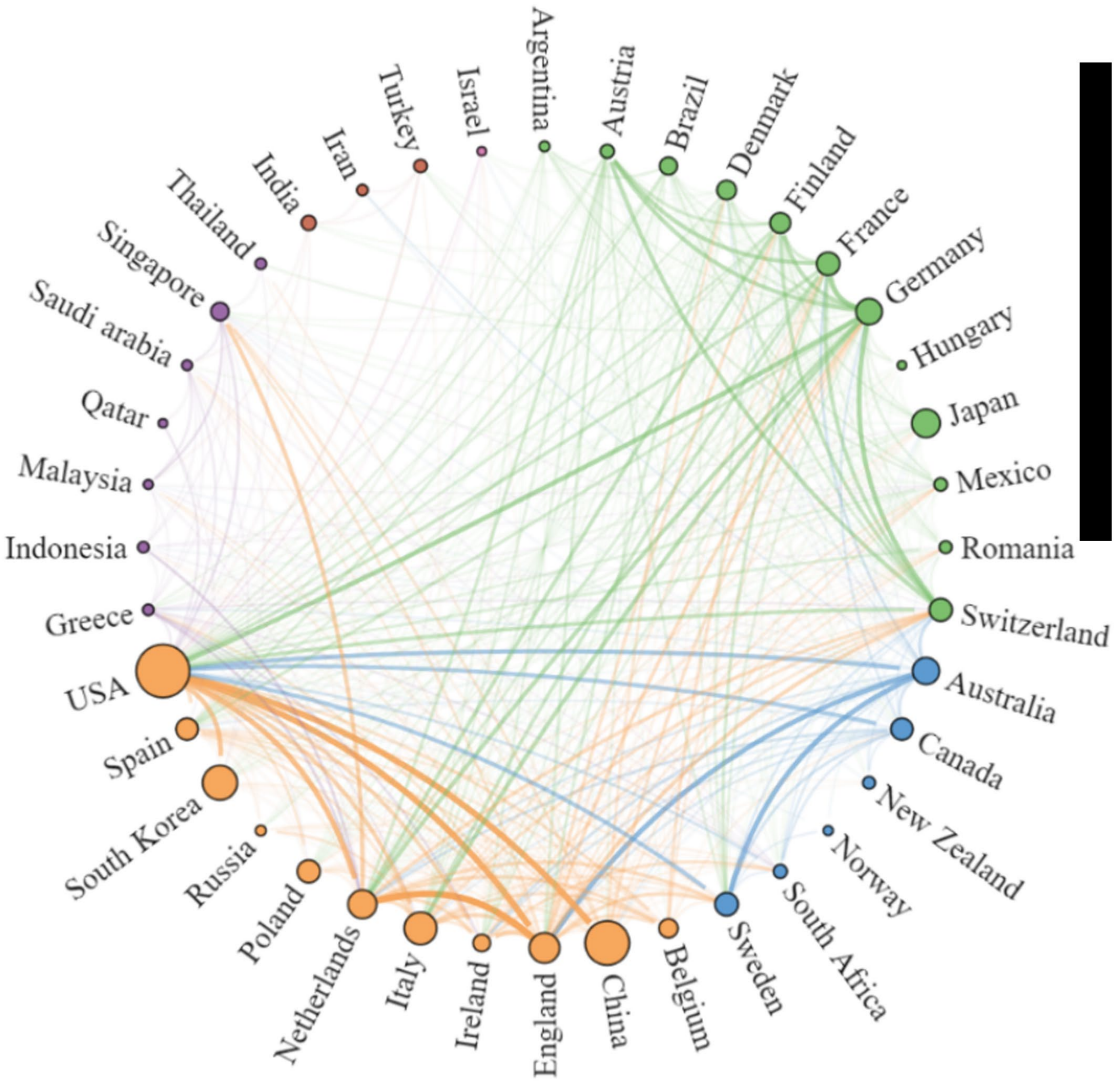
Network Map illustrating Collaborative Relationships between Countries/Regions in the research on AD and gut microbiota. To create the national cooperation map, countries that have published at least 5 articles on AD and microbiota were included. Each peripheral curve segment represents a country, and the thickness of the connection is proportional to the intensity of cooperation between countries.

Using the Scimago Graphica software, we found close cooperation between the UK and the Netherlands, China and the USA, Sweden and Australia, and Germany and Switzerland (Figure 3). The cooperation between countries can be divided into five clusters, the more papers published by countries, the larger the circle, and the closer the cooperation between countries, the thicker the connecting line, specific details are listed in Supplementary Table 1. As shown in Supplemental Table 1, the USA’s total link strength and inter-country link strength surpass those of other countries, indicating its central position and significant influence in the field of AD and gut microbiome research. It is noteworthy that although China and South Korea rank second and third in publication volume, respectively, (Table 1), they fall outside the top 10 in total link strength, indicating lower collaboration intensity with other countries.

**Figure 4:**
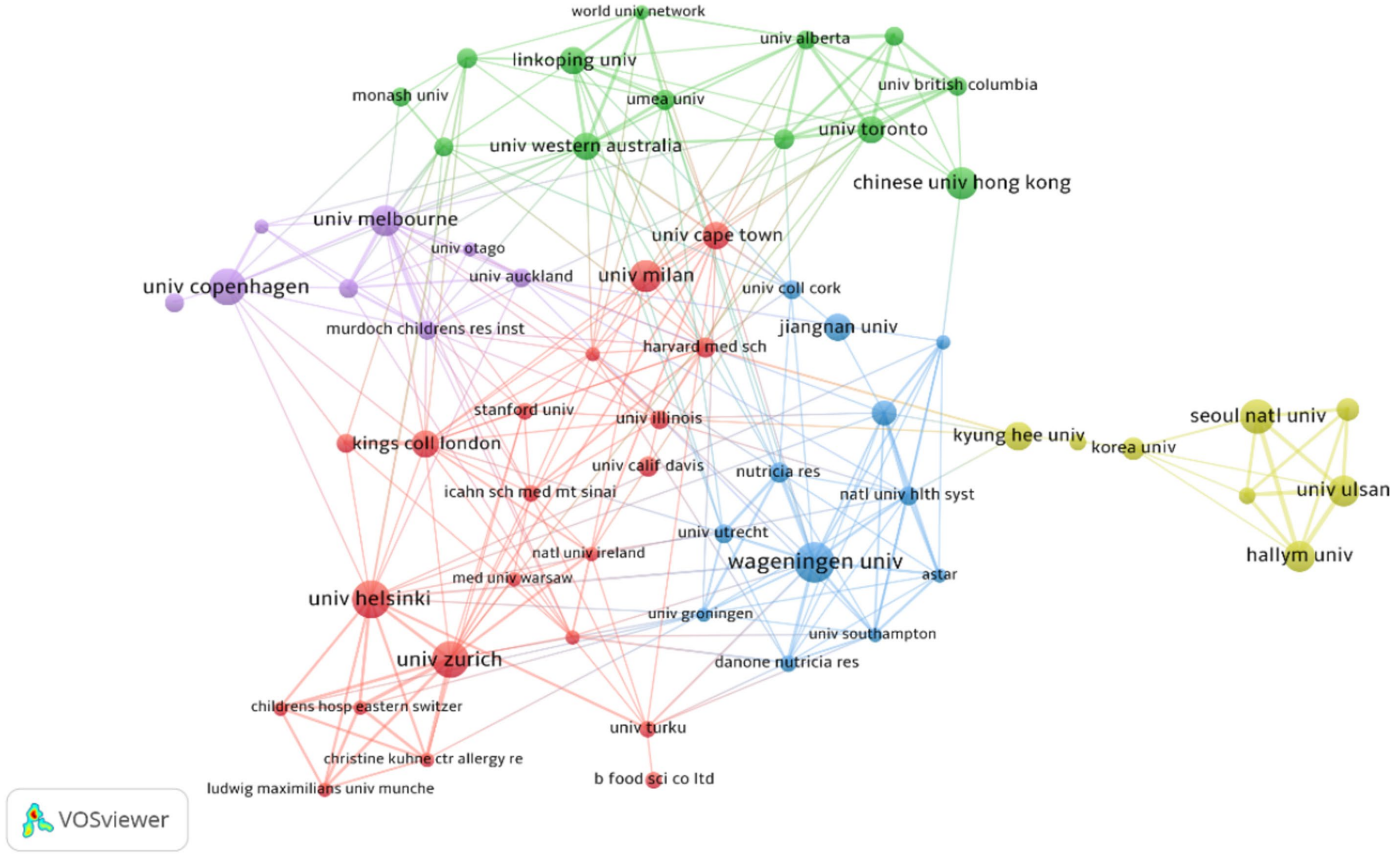
Cluster network visualization showcasing cooperation among institutions in AD and gut microbiota research. A total of 1,489 institutions published 804 articles on AD and gut microbiota related research. Different colors represent different clusters (based on the document co-citation network between institutions,institutions with high co-citation relationships are gathered together and a clustering hierarchy diagram is generated to show the relationship between institutions); the thickness of the lines connecting circles represent the intensity of cooperation between institutions; the size of the circle is positively correlated with the number of articles issued by the institution.

Table 2 presents the top 10 institutions with the highest number of published papers. Wageningen University (2.36%) ranked first, followed by University of Helsinki (2.24%), University of Copenhagen (2.11%), University of Zurich (2.11%). Percentage represents the proportion of the number of articles published by a single institution to the total number of publications. These institutions primarily originate from developed countries in Europe and South Korea, and there is no significant difference in the number of published papers among them. Figure 4 visualizes the institutions with six or more published papers, illustrating that they are divided into five clusters and actively engage in collaborations. Notably, research institutions in Korea predominantly collaborate within their own country and exhibit relatively limited international collaborations, which may be attributed to factors such as language barriers and geographical distance.

**Table 2:** The top 10 institutions contributing to publications of AD and gut microbiota.

Rank	Institutions	Number of publications	Number of citations	Citations of per article	H-index
1	Wageningen Univ	19	1,160	61.05	16
2	Univ Helsinki	18	585	32.50	11
3	Univ Copenhagen	17	574	33.76	12
4	Univ Zurich	17	1,022	60.12	11
5	Seoul Natl Univ	16	328	20.50	11
6	Chinese Univ Hong Kong	15	150	10.00	6
7	Univ Milan	15	328	21.87	10
8	Hallym Univ	14	327	23.36	9
9	Univ Melbourne	14	414	29.57	9
10	Univ Ulsan	14	562	40.14	11

### Analysis of authors and co-cited authors

**Figure 5:**
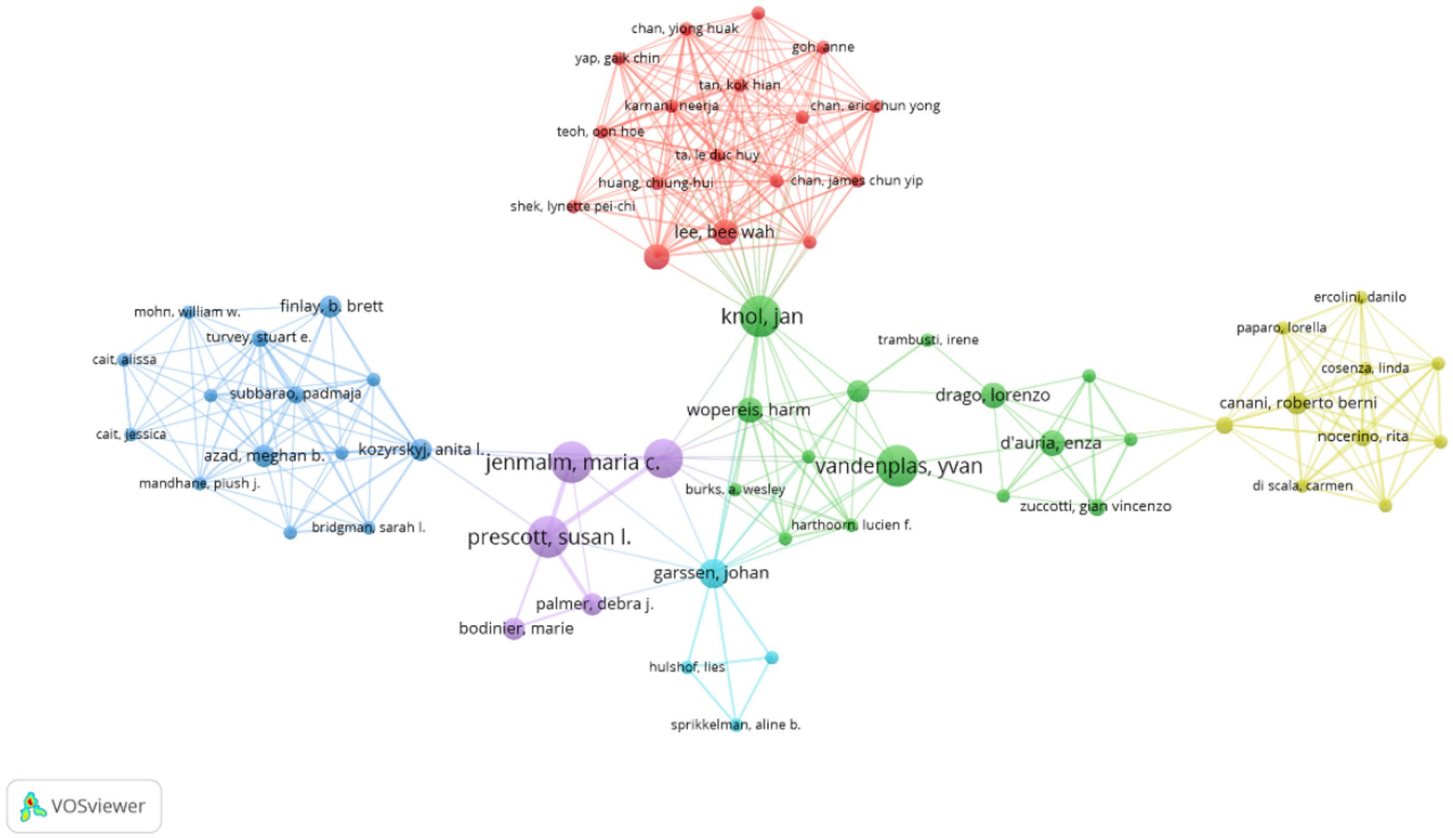
Cluster network visualization highlighting authors in AD and gut microbiota research. Visual analysis of authors was performed using VOSviewer software, and 4,329 authors published a total of 804 articles on AD and gut microbiota related research. Authors published at least 5 articles on AD and microbiota were enrolled to obtain the collaboration graph. The size of the circle is positively correlated with the number of articles published by the researcher, and the colors represent different clusters.

Using VOSviewer software, a visualization analysis was conducted on 4,329 authors who published a total of 804 articles related to AD and gut microbiota, including 366 review articles. By setting a minimum publication threshold of five articles per author, we generated a collaboration map of researchers in the AD and gut microbiota field. Authors meeting this criterion are depicted in Figure 5, where the threshold parameter limits the number of nodes. Table 3 lists the top 10 authors with the highest number of published papers. The leading author, Hong SJ (1.62%) from the University of Ulsan, is followed by Lee SY (1.24%) from Hallym University, Prescott SL (1.24%) from the University of Western Australia, and Knol J (1.24%) from Wageningen University, among others.

**Table 3:** The top 10 authors contributing to publications of AD and gut microbiota.

Rank	Authors	Number of publications	Number of citations	Citations of per article	H-index	Co-cited authors	Number of citations	Centrality
1	Hong SJ	13	522	40.15	11	Abrahamsson TR	245	0.91
2	Lee SY	10	382	38.20	9	Penders J	227	0.04
3	Prescott SL	10	552	55.20	8	Kalliomäki M	159	0.67
4	Knol J	10	750	75.00	10	Azad MB	129	0.30
5	Jenmalm MC	10	1,013	101.30	8	Dominguez-Bello MG	121	0.08
6	Vandenplas Y	10	237	23.70	4	Grice EA	120	0.56
7	West CE	9	653	72.56	8	Kong HDH	114	0.43
8	O’mahony L	9	425	47.22	8	Arrieta MC	114	0.50
9	Chen W	7	202	28.86	7	Bisgaard H	113	0.49
10	Lunjani N	7	217	31.00	7	Song H	113	0.15
11	Tochio T	7	189	27.00	5	/	/	/

The percentage represents the proportion of articles published by a single author relative to the total number of articles published. Notably, according to the citation ranking per article, Jenmalm MC from Linköping University ranks first. Jenmalm MC’s articles have received significantly more citations on average compared to those of other authors, indicating the substantial influence of their work in the field. As illustrated in Figure 5, the size of each circle correlates positively with the number of publications by the researcher, while the colors denote different clusters. The top three authors by publication count are Hong SJ, Lee SY, and Prescott SL. The authors were categorized into six clusters, each demonstrating close internal collaboration. For instance, Prescott SL and Jenmalm MC constitute one cluster, whereas Knol J and Vandenplas Y form another. Furthermore, positive collaborations were noted between different clusters, such as those between Knol J and Lee BW, as well as Kozyrskyj AL and Prescott SL. Due to the limitations of WOSCC data and the functionalities of Citaspace and VOSviewer software, distinguishing between domestic and international author collaborations remains challenging. However, analysis of Figure 4 and Supplementary Table 1 reveals that authors from South Korea and China tend to collaborate more domestically, whereas international collaborations among Western countries have significantly increased. This trend may be associated with the ease of communication in a common language.

The co-cited references were derived from a total of 25,537 authors, with 434 authors being cited 20 times or more. Table 4 illustrates that Abrahamsson TR from Linköping University was cited 245 times, ranking first, followed by Penders J (*n* = 227), Kalliomäki M (*n* = 159), Azad MB (*n* = 129), among others. Notably, Abrahamsson TR also achieved the highest centrality score of 0.91. Centrality serves as an indicator for assessing the significance of each node within a collaborative network; a higher centrality score indicates more frequent collaboration with other authors or institutions (Zhu et al., 2020). Consequently, we can conclude that the papers published by Abrahamsson TR are of high quality and have garnered substantial recognition among researchers in this field.

**Table 4:** The top 10 journals contributing to publications of AD and gut microbiota.

Rank	Journals	Number of publications	Number of citations	Citations of per article	Journal citation reports (2022)	Impact factor (2022)	H-index	Country
1	Nutrients	31	495	15.97	Q1	5.9	14	Switzerland
2	Journal of Allergy and Clinical Immunology	26	2,283	87.81	Q1	14.2	23	USA
3	Frontiers in Immunology	25	739	29.56	Q1	7.3	11	Switzerland
4	International Journal of Molecular Sciences	24	839	34.96	Q1	5.6	13	USA
5	Allergy	22	1,205	54.77	Q1	12.4	15	England
6	Microorganisms	17	420	24.71	Q2	4.5	10	Switzerland
7	Pediatric Allergy and Immunology	16	565	35.31	Q2	4.4	11	Denmark
8	Clinical and Experimental Allergy	14	1,355	96.79	Q1	6.1	12	England
9	Allergy Asthma & Immunology Research	12	368	30.67	Q2	4.4	8	Korea
10	Frontiers in Microbiology	12	1,210	100.83	Q2	5.2	8	Switzerland
11	Plos One	12	352	29.33	Q2	3.7	9	USA

### Analysis of journals, co-cited journals and co-cited references

Using VOSviewer software, we found that the academic results were published in a total of 327 journals for recognition and dissemination. Table 4 presents the top 10 journals with the largest publication volume. The journal “Nutrients” ranked first with a publication volume of 3.86%, followed by “Journal of Allergy and Clinical Immunology” (3.23%), “Frontiers in Immunology” (3.11%), and “International Journal of Molecular Sciences” (2.74%), among others. Percentages represent the proportion of the published volume of a single journal to the total published volume. These 11 journals primarily focus on the fields of allergy, immunity, microorganisms, and nutrition. Out of the 11 journals, 6 are located in Q1 (Journal Citation Reports 2022), 5 journals are located in Q2, and 7 journals have an Impact Factor (IF) of 5 or higher. These 11 journals collectively published 211 papers, accounting for 26.24% of the total. These findings indicate that the research field of AD and gut microbiota is relatively concentrated, and the obtained research results hold significant importance and are readily accepted by high-quality journals.

According to Table 4, the Journal of Allergy and Clinical Immunology had significantly more citations than other journals. Notably, the journal Frontiers in Microbiology has the highest citations per article, indicating that it publishes some highly influential articles.

Additionally, excluding the impact factor surge caused by the COVID-19 pandemic, we recalculated the impact factors of articles related to AD and gut microbiota over the past 5 years, considering two time periods: 2014–2018 and 2019–2023 (Supplementary Tables 2, 3). From Supplementary Table 3, several journals with the highest number of publications focus on allergy and immunity research, but according to Supplementary Table 2, the number of articles published in *Nutrients* ranked first in the last 5 years. In addition, the Journal Citation Reports of *Nutrients* rose from Q2 to Q1, and the impact factors also increased. This indicates that the research focus has shifted from pathogenesis to attempts at treatment, for example, AD might be treated by modulating the intestinal flora with oral prebiotics.

**Figure 6:**
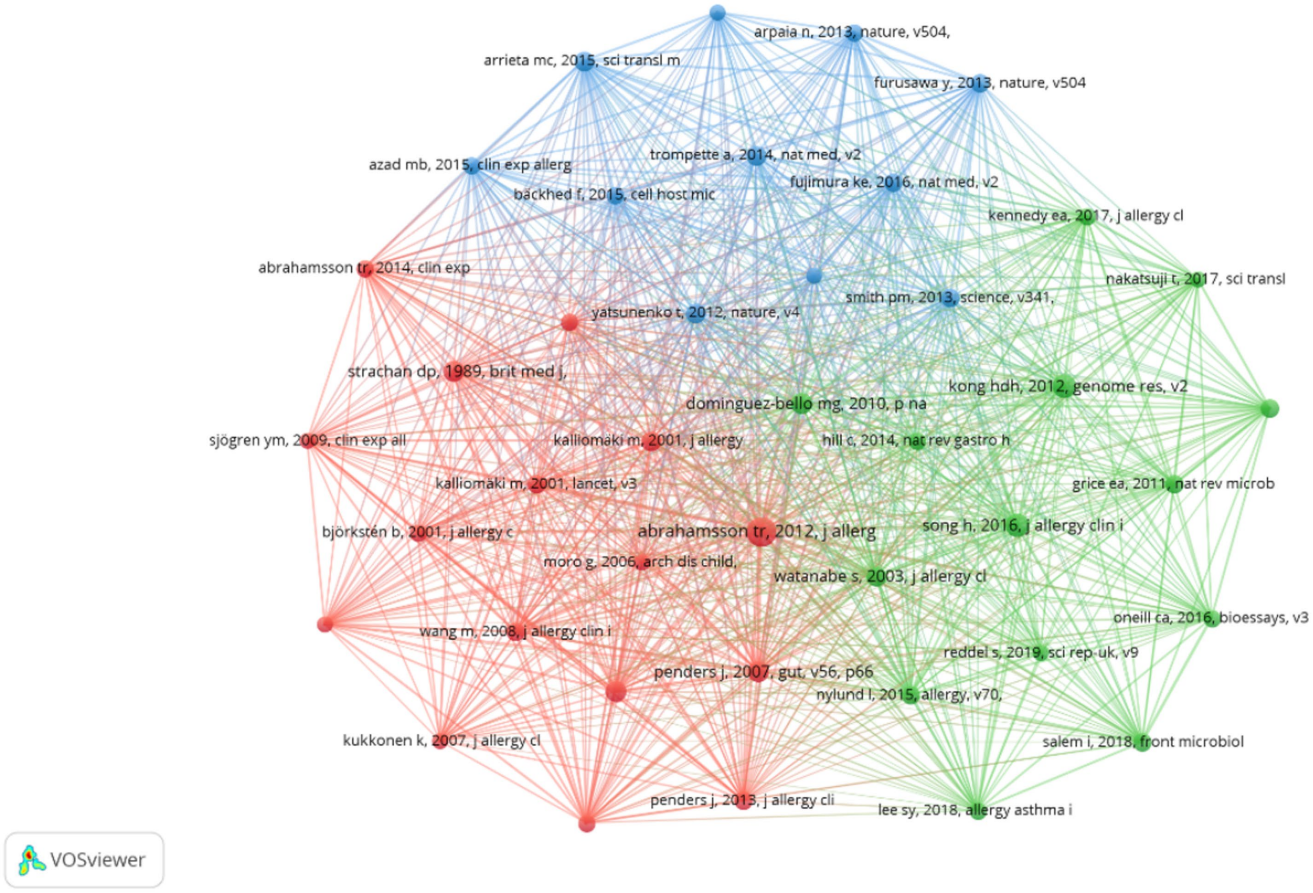
Cooperation network map of co-cited references in AD and gut microbiota research. A Cooperation network map of journals was created using CiteSpace. A total of 35,866 documents were cited, of which 41 were cited more than 50 times. The size of the circles is positively correlated with the number of citations.

Supplementary Table 4 reveals that this journal ranks first in terms of both the number of citations and centrality among co-cited journals. Additionally, Supplementary Table 5 demonstrates that five out of the top 10 co-cited references were published in the Journal of Allergy and Clinical Immunology. These findings suggest a strong preference among researchers in the field for this journal, indicating a significant inclination to submit papers to or cite articles from it. In total, the research field cited 35,866 references, with 41 of these cited 50 times or more. We visualized this data in Figure 6, which illustrates that these references are organized into three clusters characterized by active co-cited relationships, forming the foundation of the research field. Furthermore, Supplementary Table 4 indicates that, among the top 10 co-cited journals, not only specialized journals in allergy and immunology are represented, but also prestigious publications such as Nature, Science, and The Lancet. This suggests that high-quality literature from these esteemed journals is relevant to the research field and may significantly influence research progress.

The dual-map overlay of journals was created using CiteSpace software, and this map is divided into two sections: citing journals on the left and cited journals on the right. The results illustrate the positioning of research on AD and gut microbiota in relation to the primary research disciplines. Each dot on the map represents a journal, while the curves connecting the left and right sections indicate citation links. These link trajectories offer insights into the interdisciplinary relationships within the field, effectively showcasing the citation flow. The curves represent the citation paths, with thicker curves indicating a higher volume of citations (Chen, 2017). The green ovals signify the number of publications, with their size proportional to the number of published papers and authors. A longer longitudinal axis of the ovals correlates with a greater number of papers, while a longer transverse axis reflects a higher number of authors (Chen et al., 2022).

**Figure 7:**
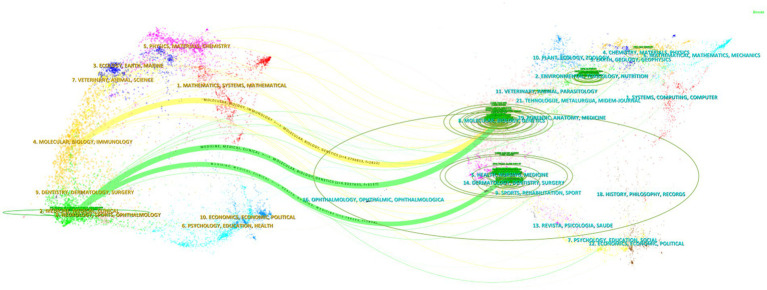
Dual-map overlay of journals in the field of AD and gut microbiota. The dual-map overlay of journals is divided into two parts: citing journals on the left and cited journals on the right. Each dot on the map represents a journal, and the curves between the left and right parts of the map indicate citation links. These link trajectories provide insights into the interdisciplinary relationships within the field, fully displaying the citation flow.

In Figure 7, the biggest and chief discipline was easily recognized as “Medicine, Medical, Clinical” in citing area and “Molecular, Biology, Genetics” in cited area. There are three main citation paths in the figure. The yellow path indicates that papers published in “Molecular, Biology, Genetics” journals are usually cited by “Molecular, Biology, Immunology” journals, and the two green paths indicate that papers published in “Medicine, Medical, Clinical” journals mainly citing “Molecular, Biology, Genetics” and “Health, Nursing, Medicine” journals. In conclusion, the study of AD and gut microbiota involves various disciplines such as medicine, immunology, molecular biology, and genetics.

### Analysis of keywords co-occurrence and bursts

**Figure 8:**
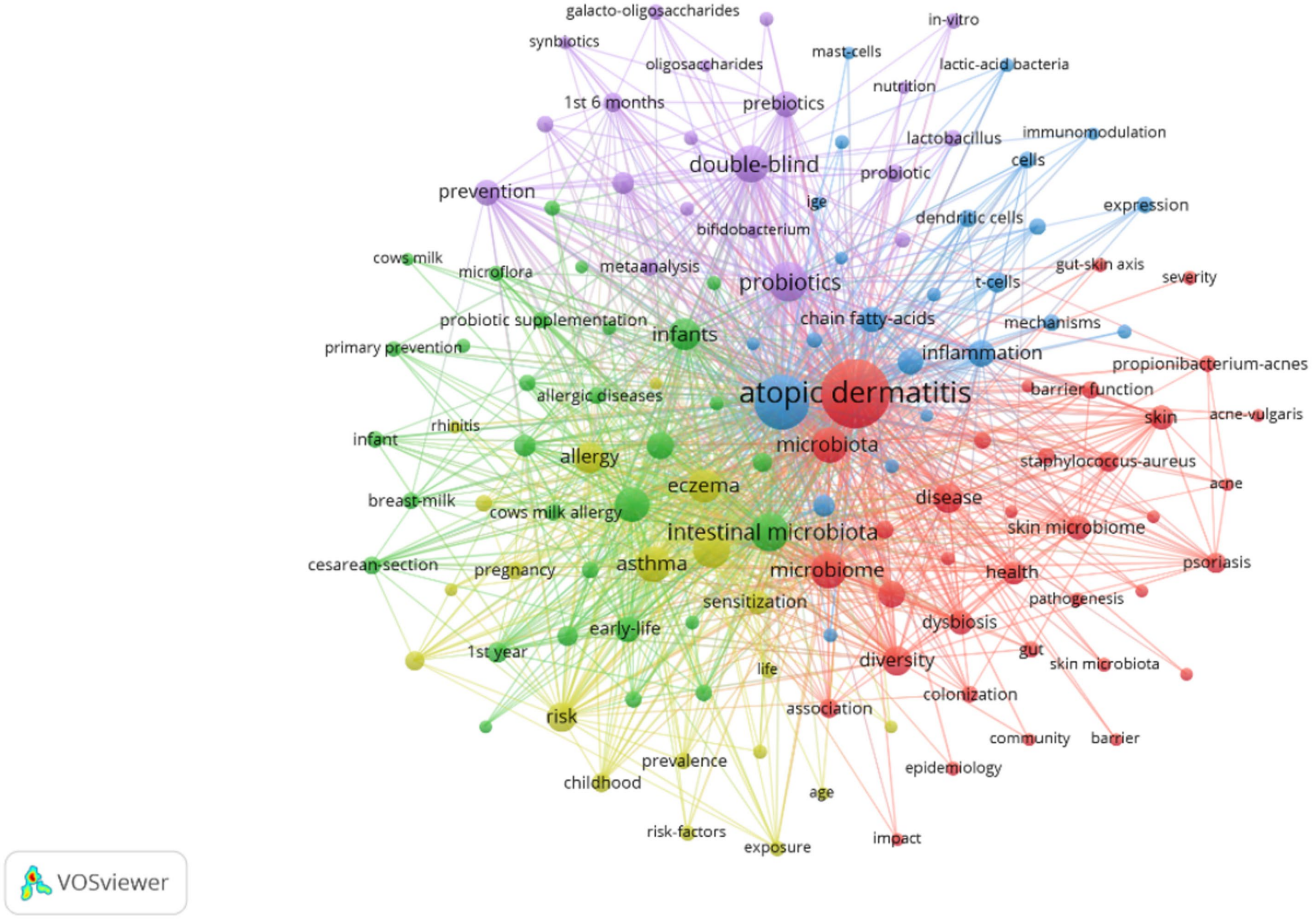
Co-occurrence network map of keywords related to AD and gut microbiota. An analysis of keyword co-occurrence clusters was performed using VOSviewer software. The minimum number of occurrences for each keyword was set to 10 times, including authors who meet the above conditions. A total of 3,001 keywords underwent cleaning, with elimination of meaningless words. After merging synonyms, 161 keywords were selected to form a visual map.

**Figure 9:**
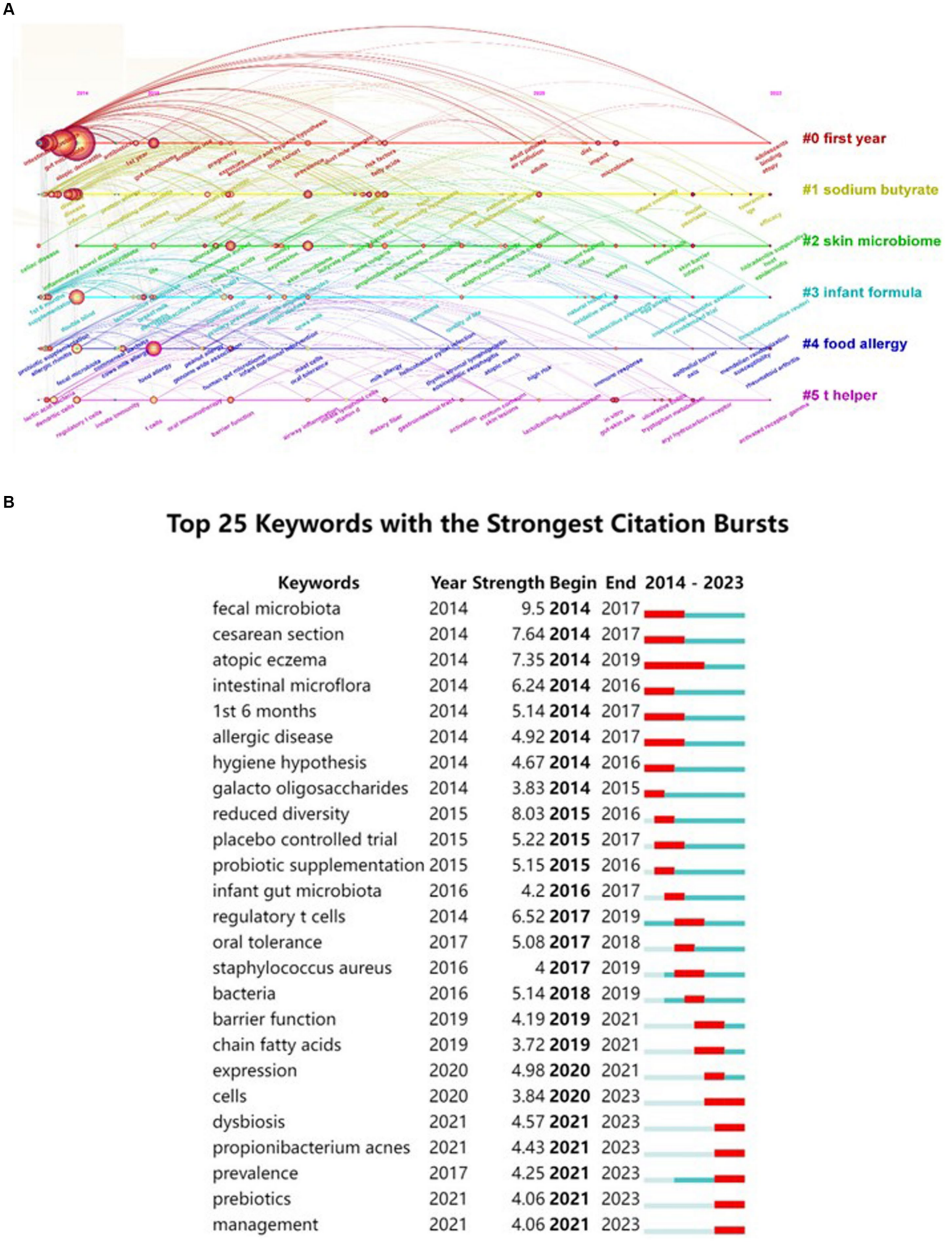
A timeline view of keywords and the top 25 keywords with the strongest citation bursts in AD and gut microbiota research was generated. **(A)** Keyword co-occurrence clusters were analyzed using VOSviewer software, with a minimum occurrence threshold of 10 times and inclusion of relevant authors. A total of 3,001 keywords underwent cleaning to remove irrelevant terms. Following synonym merging, 161 keywords were selected to construct a keyword timeline graph, where the size of each node represents the frequency of occurrence of the keyword. #1 first year means first year of life. **(B)** Subsequently, the top 25 keywords with the most significant citation bursts were identified.

Extracting keywords can grasp hot spots in the research field (Li et al., 2022). Keywords were extracted in this study, with a total of 3,001 keywords identified. Out of these, 161 keywords occurred at least 10 times. Visual analysis was conducted on these keywords, and Figure 8 shows that “atopic dermatitis” and “gut microbiota” are central to the study and closely linked to several other keywords. These keywords were categorized into 5 clusters: the red cluster represents dermatitis, the purple cluster is related to drugs and probiotics, the green cluster is associated with infants and feeding, the blue cluster represents molecular mechanisms, and the yellow cluster represents allergy. A timeline view of the keywords was generated to explore their evolution, with the size of each node on the graph determined by the frequency of occurrence. Figure 9A shows that the terms within each cluster changed over different periods. Some research areas maintained consistent intensity (#1 first year, means first year of life), some explored new directions (#5 t helper), while others gradually decreased over time (#3 infant formula, #4 food allergy). To understand the changes and frontiers in the research field, the top 25 keywords with the strongest citation bursts were counted. Figure 9B reveals that “fecal microbiota”, “Caesarean section”, and “1st 6 months” had strong citation bursts in the early stage, while in the past 3 years, researchers focused more on “dysbiosis,” “*propionibacterium acnes*,” and “prebiotics.” This suggests that regulating intestinal microecology for the treatment of AD is becoming a new research direction based on previous studies.

### Bibliometric analysis of the bibliographic coupling

**Figure 10:**
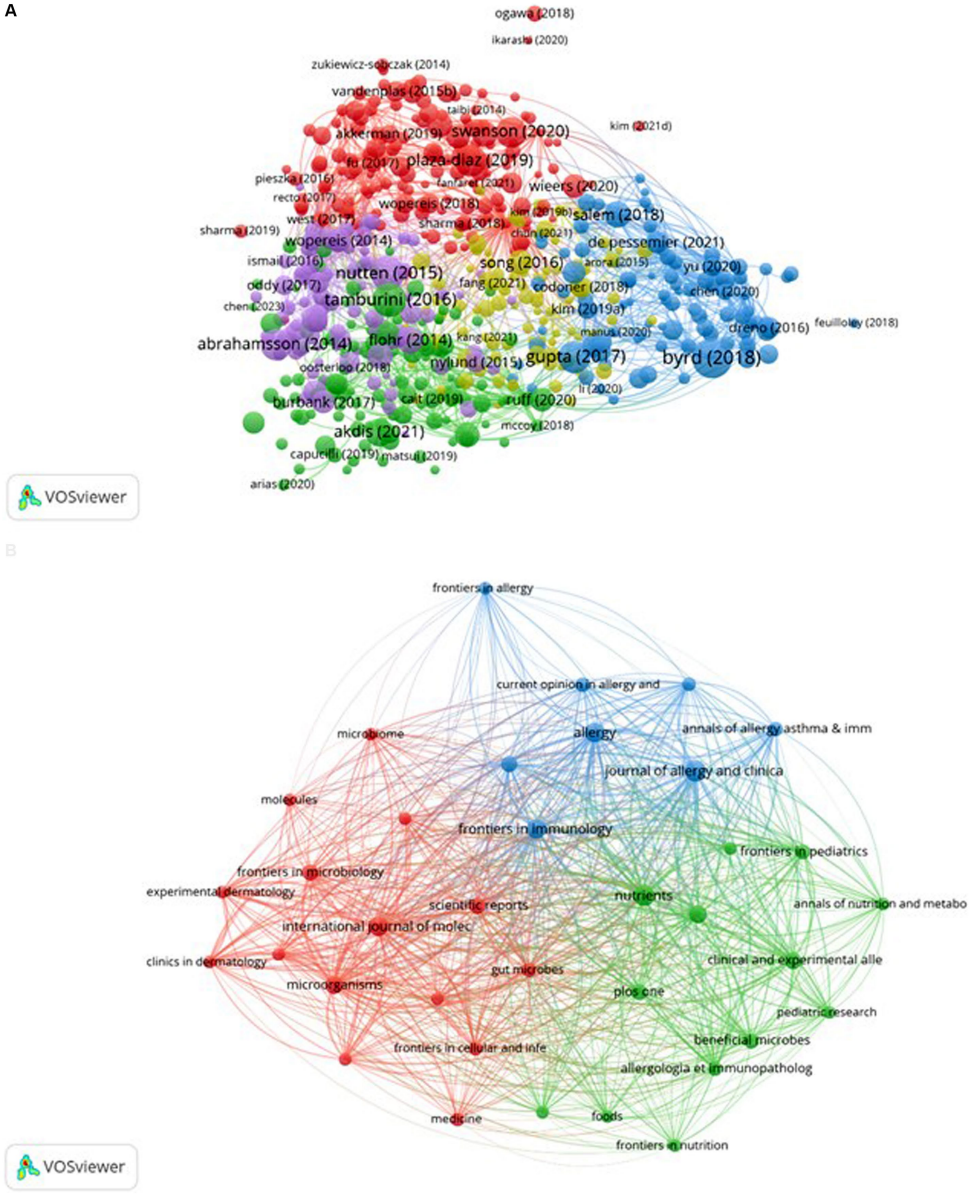
Bibliographic coupling of documents and sources. The VOSviewer software was utilized to create these maps, with different colors representing distinct research areas. The size of the circles reflects the number of co-citations, while the distance between circles signifies their correlation. Subfigure **(A)** showcases the bibliographic coupling map of documents, while subfigure **(B)** illustrates the map of sources.

The bibliographic coupling map of documents is presented in Figure 10A, revealing five distinct clusters obtained from the analysis. The literature from South Korea and China is predominantly situated within the yellow cluster. Although this cluster encompasses a greater number of items, it exhibits a lower total link strength, as indicated by fewer lines representing connection strength. This observation aligns with the results illustrated in Figure 4, suggesting that South Korea engages in less collaboration with other countries. The bibliographic coupling map of sources is depicted in Figure 10B, which identifies three clusters resulting from the analysis. The blue cluster primarily addresses allergy research, the red cluster focuses on microbiology and molecular mechanisms, while the green cluster is chiefly centered on nutrition research. These clusters encapsulate several significant research directions pertinent to AD and microbiota.

## Discussion

### General information

A total of 804 papers were included in this study, authored by 4,329 individuals from 72 countries and affiliated with 1,489 institutions. These papers were published in 327 different journals. In total, the references cited in these papers amounted to 35,866, with 279 of these references being cited more than 20 times, demonstrating a rich knowledge base and widespread interest in this research area. The analysis of publication volume revealed a rapid increase in AD and gut microbiota research starting from 2020, with an average output of 112.75 papers per year between 2020 and 2023. This surge indicates a heightened academic focus and growing recognition of the significance of this research field. However, there was a decrease in publication volume in 2023, suggesting that the research in this field has reached a mature stage. The United States had the highest number of published papers (*n* = 180), followed by China (*n* = 124). However, when considering the quality of research based on the H-index and number of citations per literature, the United States, the United Kingdom, the Netherlands, and Sweden stood out for their relatively high-quality research. Figure 3 highlights a strong collaborative network, with the United States playing a central and influential role in advancing the field of AD and gut microbiota research.

Among the analyzed institutions, Wageningen University (n = 19) emerged as the top-ranking institution, with strong publication output and minimal gaps compared to other leading institutions. Notably, active collaborations were observed among Wageningen University, University of Helsinki, and University of Melbourne, as depicted in Figure 4. However, research institutions from South Korea formed a closely-knit cluster with limited international collaboration, potentially restricting their academic growth and influence. The analysis presented in Table 4 revealed that Hong SJ from the University of Ulsan had the highest number of publications (*n* = 13), while Jenmalm MC from Linköping University led in citation metrics, and Abrahamsson TR from Linköping University was notable for high citation numbers and centrality among co-cited authors. The majority of the top 10 authors and co-cited authors hailed from developed countries in Europe, America, and South Korea, highlighting the critical role of financial investment in fostering high-quality research. These findings suggest a need for enhanced global collaboration to propel the research field forward.

As depicted in Table 3, six out of the top 10 journals with the highest number of published papers were situated in Q1 according to the Journal Citation Reports 2022, while five were in Q2, and seven had an IF of 5 points or higher, indicating significant attention towards research in this field. The Journal of Allergy and Clinical Immunology was ranked first in terms of citations and centrality within the research field. Among the subdisciplines covered by these top 10 journals, there is a notable concentration in the areas of AD and gut microbiota research, with a focus on allergy, immunity, microorganisms, and nutrition, as aligned with the dual-map overlay shown in Figure 7.

### Research trends and emerging topics

Co-cited references serve as a foundational knowledge base, reflecting core themes in a specific research field (Wang et al., 2022). Supplementary Table 5 shows that two highly co-cited reports from 2001 and 2003 identified significant variations in fecal microbiota between individuals with AD and those without the condition, underscoring the pivotal role of gut microbiota in modulating immune responses and potentially influencing the onset of atopic diseases (Kalliomäki et al., 2001; Watanabe et al., 2003). The 2001 study particularly emphasized the impact of neonatal gut microbiota on atopic disease susceptibility, highlighting the importance of early microbial colonization patterns (Kalliomäki et al., 2001).

The work of Lv H, et al. underscores the rise of allergic diseases due to microbiota dysbiosis, documenting significant research output from 2002 to 2021. Key topics include the influence of *Staphylococcus aureus*, the broader microbiome, and the roles of short-chain fatty acids (SCFAs) and long-chain polyunsaturated fatty acids (LCPUFAs) in allergies. The article also discusses emerging topics, such as mechanistic insights and potential therapeutic approaches targeting microbiota-related pathways (Kim et al., 2021). Kim D, et al. provide a comprehensive overview of AD research trends, noting an increase in studies since 1990, particularly focusing on asthma, food allergies, and the skin barrier. The skin microbiome is highlighted as a crucial factor in AD development, with persistent themes such as the atopic march, quality of life in AD management, and the interplay between the skin microbiome and AD pathology (Lv et al., 2022).

Incorporating gut microbiota research into the study of AD aligns with broader trends in allergic disease research. While Lv H, et al. emphasized the role of gut microbiota, Kim D, et al. primarily focused on the skin microbiome, indicating a need for an integrative approach that considers both ecosystems. The surge in studies post-2010, particularly those among the top 10 co-cited references, underscores the growing recognition of gut microbiota’s role in allergic diseases (Abrahamsson et al., 2012; Dominguez-Bello et al., 2010; Bisgaard et al., 2011; Kong et al., 2012; Arrieta et al., 2015; Song et al., 2016).

For instance, Abrahamsson et al.’s (2014) study linked low total intestinal flora diversity in infants to asthma in 7-year-olds, a finding cited 496 times, significantly influencing the field (Arrieta et al., 2015). Similarly, a 2016 study utilized 16S rRNA gene and metagenomic sequence analysis to explore the gut microbiota in AD patients, suggesting a complex interplay between specific gut microbiota profiles and AD progression (Song et al., 2016).

The result suggests that feedback interactions between the enrichment of a particular subspecies of *Faecalibacterium prausnitzii* and dysregulation of gut epithelial inflammation might be related to the progression of AD. The authors speculate that dysbiosis in *F prausnitzii* impairs the intestinal barrier, which ultimately leads to aberrant TH2 type immune response of skin to allergens. This genetic study provides a pivotal foundation for exploring the mechanisms by which gut microbiota influence human health, with a particular focus on AD in infants. The continued interest in the “#5 T helper” cluster, as depicted in Figure 9A, underscores the significance of understanding the immune pathways involved in AD. Future research should prioritize elucidating these mechanisms to develop targeted therapeutic strategies (Fang et al., 2022; Xie et al., 2023).

Keywords with citation bursts can serve as indicators of evolving research priorities. According to Figure 9B, “caesarean section” and “1st 6 months” emerged as notable research hotspots from 2014 to 2017. A 2010 study established that infants delivered vaginally acquire gut microbiota resembling their mothers’ vaginal flora, predominantly *Lactobacillus, Prevotella*, and *Sneathia* spp., while cesarean-delivered infants exhibit microbiota akin to skin flora, including *Staphylococcus*, *Propionibacterium*, and *Corynebacterium* spp. (Dominguez-Bello et al., 2010). This finding set the stage for investigating how delivery mode influences infant health. Subsequent research in 2014 linked that cesarean section with delayed colonization of *Bifidobacteria* and *Bacteroidetes*, noting an association between decreased *Lactobacillus* and *Bifidobacterium* levels and the development of allergic diseases in infants (Hesla et al., 2014). This association has been consistently supported by further studies, indicating that altered gut microbiota colonization in cesarean -born children as a potential risk factor for allergic diseases (Papathoma et al., 2016; Gerlich et al., 2018; Cheung et al., 2023; Galazzo et al., 2020).

Since 2017 there have been significant citation bursts related to “oral tolerance” and “short-chain fatty acids”. For instance a study by Nowak-Węgrzyn et al. suggested that introducing peanuts into the diets of infants aged 4–6 months who have egg allergies or severe eczema could promote oral tolerance and reduce the risk of eczema development (Nowak-Węgrzyn and Chatchatee, 2017). Additionally regulating gut microbiota through prebiotics and probiotics has emerged as a promising avenue for inducing intestinal tolerance. Another study explored the correlation between gut microbiota composition short-chain fatty acids (SCFAs) and AD in 6-month-old infants revealing a positive correlation between the abundance of *Streptococcus* and a negative correlation with *Clostridium*. Infants with transient AD were found to have lower levels of butyrate and valerate potentially impairing the intestinal epithelial barrier (Park et al., 2020).

In the past 3 years, there has been a surge in interest in “Dysbiosis,” “prebiotics,” and “management,” suggesting that the treatment of AD by regulating intestinal microecology has become a focal point of research (Fanfaret et al., 2021). For example, Wopereis et al. demonstrated that infant formula enriched with specific prebiotics could mimic the beneficial effects of breast milk on intestinal flora (Wopereis et al., 2018). Another study utilized an AD-like mouse model to test the therapeutic effects of orally administered prebiotics, finding a correlation between prebiotic intake and favorable changes in gut microbiota (Kang et al., 2023). Moreover, a meta-analysis highlighted that combining probiotics with topical corticosteroids can effectively alleviate AD symptoms, suggesting their potential as an adjunctive treatment (Xue et al., 2023). Further studies have characterized the pro-inflammatory gut microbiota in children with food allergies, often rich in genes related to bacterial lipopolysaccharide production and urease enzyme activity. Notably, active *Helicobacter pylori* was identified as a predominant pathogen in allergic children, potentially contributing to immune dysregulation and altered food tolerance through interactions with gut microbiota. Emerging management strategies, including probiotics, prebiotics, fecal microbiota transplantation, postbiotics, and synthetic prebiotics, hold promise for modulating the gut microbiota and immune response in food allergies (Kim and Kim, 2019; Vieira-Silva et al., 2016; Bunyavanich and Berin, 2019).

However, the effectiveness of probiotics/prebiotics/symbiotics in regulating intestinal flora for the prevention and treatment of allergic diseases remains controversial (Fiocchi et al., 2022; Petersen et al., 2019). Clinicians may consider using these modulators as adjunct treatment for preventing AD, their utility for other allergic conditions requires further validation. Therefore, further evidence is necessary to support the use of microbial ecological agents as alternative treatments for AD.

### Limitations

This study has common deficiencies in bibliometrics. Although we want to fully include the papers in the research field as much as possible, due to the limitation of the software, we used the WOSCC database for retrieval, which may lead to incomplete included papers and certain bias. Secondly, we limit the language to English and the type of literature to articles and reviews, which may not be rigorous enough. Finally, we merged keywords according to the standardized steps of bibliometrics, such as the names of authors and institutions, but because the same name may have many expressions, the same author or institution may not be fully merged, which affected the accuracy of the research results.

## Conclusion

In the past 10 years, more and more papers have been published in the research field, indicating that the research on AD and gut microbiota has become more and more popular. With the continuous deepening of research, researchers have analyzed the correlation between AD and gut microbiota from the early stage, and gradually paid attention to the mechanism of gut microbiota and the efficacy of microbial ecological agents. “T helper” and “prebiotics” represent different research hotspots. In conclusion, as the first bibliometric study to analyze AD and gut microbiota, this study reviews the research trends in the past 10 years, analyzes the research hotspots and future research directions, which can provide references for further research in this field.

## Data Availability

The raw data supporting the conclusions of this article will be made available by the authors, without undue reservation.
