# Supplementary material for: Mapping the relationship between atopic dermatitis and gut microbiota: a bibliometric analysis, 2014–2023
Source: Front Microbiol. 2024 Sep 4;15:1400657. doi: 10.3389/fmicb.2024.1400657 (PMC11408322; doi:10.3389/fmicb.2024.1400657)
Supplement: Supplementary file 1 [file Data_Sheet_1.docx]

**SUPPLEMENTARY TABLE 1 The top 10 countries contributing to publications of AD and gut microbiota**

| Rank | Country | Number of publications | Number of citations | Citations of per article | H-index |
| --- | --- | --- | --- | --- | --- |
| 1 | USA | 180 | 9016 | 50.09 | 47 |
| 2 | China | 124 | 2332 | 18.81 | 25 |
| 3 | South Korea | 76 | 1575 | 20.72 | 20 |
| 4 | Italy | 67 | 2062 | 30.78 | 24 |
| 5 | England | 56 | 2521 | 45.02 | 25 |
| 6 | Netherlands | 52 | 2013 | 38.71 | 25 |
| 7 | Japan | 51 | 914 | 17.92 | 16 |
| 8 | Australia | 46 | 1724 | 37.48 | 23 |
| 9 | Germany | 43 | 1522 | 35.40 | 20 |
| 10 | Sweden | 34 | 2155 | 63.38 | 25 |
| 11 | France | 34 | 1275 | 37.50 | 16 |

**SUPPLEMENTARY TABLE 2 The top 10 institutions contributing to publications of AD and gut microbiota**

| Rank | Institutions | Number of publications | Number of citations | Citations of per article | H-index |
| --- | --- | --- | --- | --- | --- |
| 1 | Wageningen Univ | 19 | 1160 | 61.05 | 16 |
| 2 | Univ Helsinki | 18 | 585 | 32.50 | 11 |
| 3 | Univ Copenhagen | 17 | 574 | 33.76 | 12 |
| 4 | Univ Zurich | 17 | 1022 | 60.12 | 11 |
| 5 | Seoul Natl Univ | 16 | 328 | 20.50 | 11 |
| 6 | Chinese Univ Hong Kong | 15 | 150 | 10.00 | 6 |
| 7 | Univ Milan | 15 | 328 | 21.87 | 10 |
| 8 | Hallym Univ | 14 | 327 | 23.36 | 9 |
| 9 | Univ Melbourne | 14 | 414 | 29.57 | 9 |
| 10 | Univ Ulsan | 14 | 562 | 40.14 | 11 |

**SUPPLEMENTARY TABLE 3 The top 10 authors contributing to publications of AD and gut microbiota**

| Rank | Authors | Number of publications | Number of citations | Citations of per article | H-index | Co-cited authors | Number of citations | Centrality |
| --- | --- | --- | --- | --- | --- | --- | --- | --- |
| 1 | Hong SJ | 13 | 522 | 40.15 | 11 | Abrahamsson TR | 245 | 0.91 |
| 2 | Lee SY | 10 | 382 | 38.20 | 9 | Penders J | 227 | 0.04 |
| 3 | Prescott SL | 10 | 552 | 55.20 | 8 | Kalliomäki M | 159 | 0.67 |
| 4 | Knol J | 10 | 750 | 75.00 | 10 | Azad MB | 129 | 0.30 |
| 5 | Jenmalm MC | 10 | 1013 | 101.30 | 8 | Dominguez-Bello MG | 121 | 0.08 |
| 6 | Vandenplas Y | 10 | 237 | 23.70 | 4 | Grice EA | 120 | 0.56 |
| 7 | West CE | 9 | 653 | 72.56 | 8 | Kong HDH | 114 | 0.43 |
| 8 | O'mahony L | 9 | 425 | 47.22 | 8 | Arrieta MC | 114 | 0.50 |
| 9 | Chen W | 7 | 202 | 28.86 | 7 | Bisgaard H | 113 | 0.49 |
| 10 | Lunjani N | 7 | 217 | 31.00 | 7 | Song H | 113 | 0.15 |
| 11 | Tochio T | 7 | 189 | 27.00 | 5 | / | / | / |

**SUPPLEMENTARY TABLE 4 The top 10 journals contributing to publications of AD and gut microbiota**

| Rank | Journals | Number of publications | Number of citations | Citations of per article | Journal citation reports (2022) | Impact factor (2022) | H-index | Country |
| --- | --- | --- | --- | --- | --- | --- | --- | --- |
| 1 | *Nutrients* | 31 | 495 | 15.97 | Q1 | 5.9 | 14 | Switzerland |
| 2 | *Journal of Allergy and Clinical Immunology* | 26 | 2283 | 87.81 | Q1 | 14.2 | 23 | USA |
| 3 | *Frontiers in Immunology* | 25 | 739 | 29.56 | Q1 | 7.3 | 11 | Switzerland |
| 4 | *International Journal of Molecular Sciences* | 24 | 839 | 34.96 | Q1 | 5.6 | 13 | USA |
| 5 | *Allergy* | 22 | 1205 | 54.77 | Q1 | 12.4 | 15 | England |
| 6 | *Microorganisms* | 17 | 420 | 24.71 | Q2 | 4.5 | 10 | Switzerland |
| 7 | *Pediatric Allergy and Immunology* | 16 | 565 | 35.31 | Q2 | 4.4 | 11 | Denmark |
| 8 | *Clinical and Experimental Allergy* | 14 | 1355 | 96.79 | Q1 | 6.1 | 12 | England |
| 9 | *Allergy Asthma & Immunology Research* | 12 | 368 | 30.67 | Q2 | 4.4 | 8 | Korea |
| 10 | *Frontiers in Microbiology* | 12 | 1210 | 100.83 | Q2 | 5.2 | 8 | Switzerland |
| 11 | *Plos One* | 12 | 352 | 29.33 | Q2 | 3.7 | 9 | USA |

**SUPPLEMENTARY TABLE 5 The top 10 co-cited journals of AD and gut microbiota**

| Rank | Co-cited journals | Number of citations | Centrality |
| --- | --- | --- | --- |
| 1 | *Journal of Allergy and Clinical Immunology* | 699 | 0.74 |
| 2 | *Plos One* | 552 | 0.21 |
| 3 | *Clinical and Experimental Allergy* | 531 | 0.21 |
| 4 | *Allergy* | 501 | 0.03 |
| 5 | *Nature* | 461 | 0.58 |
| 6 | *Science* | 401 | 0.04 |
| 7 | *Proceedings of the National Academy of Sciences of the United States of America* | 400 | 0.21 |
| 8 | *Pediatric Allergy And Immunology* | 394 | 0.14 |
| 9 | *Lancet* | 354 | 0.01 |
| 10 | *Scientific Reports* | 346 | 0 |

**SUPPLEMENTARY TABLE 6 The top 10 co-cited references of AD and gut microbiota**

| Rank | Title | Journal | Author | Year | Number of co-citations | Number of co-citations per year |
| --- | --- | --- | --- | --- | --- | --- |
| 1 | Low diversity of the gut microbiota in infants with atopic eczema | *Journal of Allergy and Clinical Immunology* | Abrahamsson TR | 2012 | 191 | 15.92 |
| 2 | Temporal shifts in the skin microbiome associated with disease flares and treatment in children with atopic dermatitis | *Genome Research* | Kong HDH | 2012 | 119 | 9.92 |
| 3 | Faecalibacterium prausnitzii subspecies–level dysbiosis in the human gut microbiome underlying atopic dermatitis | *Journal of Allergy and Clinical Immunology* | Song H | 2016 | 117 | 14.63 |
| 4 | Gut microbiota composition and development of atopic manifestations in infancy: the KOALA Birth Cohort Study | *Gut* | Penders J | 2007 | 101 | 5.94 |
| 5 | Delivery mode shapes the acquisition and structure of the initial microbiota across multiple body habitats in newborns | *Proceedings of the National Academy of Sciences of the United States of America* | Dominguez-Bello MG | 2010 | 101 | 7.21 |
| 6 | Hay fever, hygiene, and household size | *BMJ Clinical Research* | Strachan DP | 1989 | 96 | 3.84 |
| 7 | Reduced diversity of the intestinal microbiota during infancy is associated with increased risk of allergic disease at school age | *Journal of Allergy and Clinical Immunology* | Bisgaard H | 2011 | 95 | 7.31 |
| 8 | Differences in fecal microflora between patients with atopic dermatitis and healthy control subjects | *Journal of Allergy and Clinical Immunology* | Watanabe S | 2003 | 89 | 4.24 |
| 9 | Early infancy microbial and metabolic alterations affect risk of childhood asthma | *Science Translational Medicine* | Arrieta MC | 2015 | 86 | 9.56 |
| 10 | Distinct patterns of neonatal gut microflora in infants in whom atopy was and was not developing | *Journal of Allergy and Clinical Immunology* | Kalliomäki M | 2001 | 83 | 3.61 |
